# Supplementary material for: Are carbon emissions trading and green financial instruments synergistic? -Comprehensive quantitative research based on content analysis
Source: PLoS One. 2024 Mar 7;19(3):e0298601. doi: 10.1371/journal.pone.0298601 (PMC10919601; doi:10.1371/journal.pone.0298601)
Supplement: S1 Data — (ZIP) [file pone.0298601.s001.zip › Data/地级市Z_F工作报告.zip!/║┌┴·╜¡/╝╤─╛╦╣╩╨/└┤╫╘╣1⁄2╓┌║┼í╛┬φ┐╦╩2╛▌═°í┐_2018.docx]

各位代表：

现在，我代表市人民政府，向大会报告工作，请予审议，并请市政协委员提出意见。

一、2017年工作回顾

过去的一年，在省委、省政府和市委的正确领导下，我们团结带领全市人民，攻坚克难，开拓奋进，较好地完成市十六届人大一次会议确定的目标任务，保持了经济平稳健康发展、社会和谐稳定。2017年，预计全市地区生产总值同比（下同）增长6.3%；固定资产投资增长7.5%；一般公共财政预算收入增长1.6%；社会消费品零售总额增长9.4%。

――强基础促增收，现代农业加快发展。以供给侧结构性改革为重点，加快调结构、增效益、进市场，农业现代化建设水平不断提高。新增水稻37.5万亩、大豆195.5万亩，调减玉米261.3万亩，新增蔬菜6.29万亩，食用菌生产规模达到4亿袋，落实农业“三减”面积322万亩，国家级绿色食品原料标准化生产基地达到840万亩，“互联网+农业”高标准示范基地发展到110处，锦西灌区等大型灌区新建续建工程扎实推进，粮食生产实现“十四连丰”。实施“两牛一猪”工程，盛世凯达万头奶牛养殖项目建成投产，双兆猪业20万头生猪养殖项目一期工程完工，有力推动粮变肉、草变乳过腹增值。抓好“粮头食尾”“农头工尾”，加快一二三产融合发展，东极寒地红树莓深加工等农业精深加工项目相继开工投产，稻海田园综合体等设施农业、体验农业、观光农业项目加快建设，市级规模以上龙头企业达到190户，中高端绿色有机农产品供给能力不断提高。开展 “品牌农业中国行―佳木斯大米走进佛山”等系列活动，启动中国大米产业展览交易基地立项，“佳木斯大米”进入省农产品地理标志十大区域品牌榜，“佳木斯大豆”地理标志成功获批，全市地理标志总数达到14个，“生态米都”区域品牌影响力不断增强。具有农业特色的国家高新技术产业开发区创建工作全面启动。

――转方式调结构，工业经济企稳回升。加快工业转型升级步伐，工业经济走出低迷，预计规上工业增加值增长5%。实施培育规上工业企业专项行动，新增规上企业24户。组建农机创新联盟，常发佳联等15户企业完成技术改造，海瑞特特种工程塑料项目列入《中国制造2025工业企业技术改造升级导向计划》，装备制造、食品、建材等传统产业改造升级不断加快。深入实施创新驱动发展战略，成功举办“创新驱动助力工程”院士专家佳木斯行活动，建立企业院士工作站、科学家工作室、博士后工作站5个，签订技术合作协议10项，与中国科学院长春分院建立战略合作关系，“三江工业云”上线运行，佳电股份10KW高压核电电机、润特科技可控可降解地膜等30个重点新产品投产或试投产。组织开展科技型企业三年行动计划，新增科技型中小企业340户，新增国家级高新技术企业7户，产业技术创新战略联盟总数达到8家，科技企业孵化器和众创空间达到14家，复制大学生创业社区6个，新建创业创新孵化基地15个。高新区内农机、食品、包装等特色产业园承载功能不断提升。

――优服务提水平，城市功能持续完善。服务业对经济增长贡献率不断提高。统筹推进大型商业综合体、购物中心和专业大市场建设，万达城市广场等大型城市综合体实现稳定运营。联动开展农机展洽会、文博会、三江国际旅游节、稻米节“四大节会”，进一步提升了城市形象。与中国网库、京东商城、阿里巴巴等企业的电商合作项目相继落地，电子商务产业园已有112家企业入驻运营，农业电子商务创业基地提档升级，佳美网等15个电商平台建成运营，汤原、桦川获批国家级电子商务进农村综合示范县。启动全域旅游创建，组建龙江东部旅游产业联盟，重点打造 “追随总书记脚步，赫乡民俗体验游”等一批精品线路，汤原大亮子河森林公园、郊区大来岗风景区被确定为2017全国旅游优选项目，全年接待国内外游客555.5万人次，实现旅游收入23.3亿元。佳木斯机场新增航线4条，年度旅客吞吐量达到66万人次。加强城市规划、建设和管理，新建续建市区路桥工程11项，4座城市立交桥建成通车，数字化城管平台投入使用，交通综合整治深入实施，城市综合承载功能持续增强。注重黑土地、森林、湿地保护，松花江沿岸砂场综合整治取得明显成效，新建美丽乡村121个。单位GDP能耗、化学需氧量和二氧化硫减排量均完成年度目标。

――上项目扩投资，发展后劲日益增强。全市产业项目完成投资450亿元，增长9.8%，开复工亿元以上产业项目119项，泉林生态农业秸秆综合利用一期项目、象屿金谷粮食仓储和玉米淀粉加工项目建成投产，东北制药10万吨山梨醇等项目签约落地，大项目辐射带动效应日益显现。“央企佳木斯对接行动”取得阶段性成果，共签订战略框架合作协议9项，达成合作意向33项，华润医疗物流配送、康复医养中心等项目开工建设，玉米燃料乙醇、寒地浆果精深加工、佳纸综合改造利用等项目前期工作进展顺利。加快推进铁路、水利、公路等重大基础设施建设，中俄同江跨江铁路大桥中方一侧基本完工，哈佳快速铁路全线铺轨贯通，佳木斯站综合改造项目南站房、南广场建成投入使用，牡佳客运专线全面开工，大来旅游公路竣工通车，松花江二桥和佳木斯机场迁建前期工作有序推进。

――抓改革促开放，新动能不断激发。坚持以改革破难题、以改革增活力、以改革促发展。“放管服”改革纵深推进，取消行政权力事项64项， “双随机一公开”制度实现全覆盖，政务及公共服务云平台建成运行，网上平台与实体大厅加快融合。现代农业综合配套改革深入推进，全市各类新型经营主体发展到7935个，流转土地面积515万亩，占集体耕地的42.6%，土地承包经营权确权登记实测全面完成；启动农村集体产权制度改革省级试点村10个、市级试点村11个、国家试点县1个；同江农村信用社成为我市首家改制成功的农村商业银行，桦南在全市率先成立鸿源农村资金互助社并挂牌运行。生态文明体制改革扎实推进，河长制改革全面推行，市县乡村四级河长组织体系和工作机制基本建立，全域性不动产登记正式启动。城市公立医院综合改革稳步实施，药品加成全部取消（中药饮片除外）。在全市12个乡镇开展强乡扩权改革试点，全国乡镇政府能力建设研讨会在桦南成功召开。深化对外开放合作，同江口岸东部正式作业区上报国务院待批，佳天国际俄罗斯商品批发大卖场和食品加工园区项目全面启动，抚远冷链物流产业园、莽吉塔铁路货场、江海石油港等项目加快建设，新远东公用型保税仓库投入运营，同江丰林达循环经济工业园等对俄合作园区承载功能不断增强，全市新增外贸主体52家，22家传统企业进出口额实现倍增。与广东省中山市开展对口合作，率先启动人员互派挂职。

――惠民生增福祉，社会保持和谐稳定。克服财政收支矛盾，全市各级财政用于民生支出165亿元，占公共财政预算支出66%。把脱贫攻坚作为第一民生工程，坚持精准扶贫、精准脱贫，扎实开展“回头看”工作，以产业扶贫为根本，以金融扶贫为重点，落实落靠各项扶贫措施，切实提升“三保障”和饮水安全水平，全市实现年度精准脱贫5708户、12160人。以创业带就业，城镇新增就业4.51万人次，城镇登记失业率控制在4.05%，零就业家庭保持动态为零，预计城乡居民人均可支配收入分别增长6.5%和5.5%。社保覆盖面持续扩大，各类社会保险提标扩面，城乡低保、救灾救济等社会救助体系不断完善，养老服务体系初步建立。新建农村公路1040公里，改造供热管网33.9公里、供水管线42.2公里，新建燃气管线60.3公里，新增燃气用户1.6万户。保障性安居工程超额完成目标任务，棚户区改造12109套，分配公租房1925户，发放廉租房租金补贴11025户，争取补助资金5119万元。维修老旧小区楼房158栋，改造农村危房8912户。新建公交停车泊位100个，100台新能源电动公交车上线运营。拆除各类违章建筑12万平方米，城市绿化覆盖率达到41.61%。10蒸吨及以下燃煤小锅炉淘汰率95.6%，淘汰黄标车3680台。义务教育“两免一补”政策实现城乡全覆盖，同江等6个县（市）区通过义务教育发展基本均衡国家督导验收。健康佳木斯建设蓬勃开展，分级诊疗逐步推广，医联体建设步伐加快，与国内知名医院组建医疗专科29个，贫困村卫生室标准化建设全部达标，开展健康讲座1400余场。市级“三馆”、县级“两馆”提档升级，举办各种文艺演出1200余场，富锦万人大秧歌成功挑战吉尼斯世界记录。竞技体育水平大幅提高，全市体育人口由32%增加到36%。深化矛盾纠纷排查化解工作，专人专班推进回迁办证信访积案化解，解决6965户回迁和70117户办照等历史遗留问题。深入开展平安建设，“雪亮工程”扎实推进，连续两年荣获“全国最安全城市”称号。食品药品监管和安全生产工作不断加强，国家食品安全示范城市创建工作扎实推进，连续六年荣获“中国十佳食品安全城”称号。

国防动员、人民防空、民兵预备役、双拥、优抚安置等工作进一步加强，外事侨务、民族宗教、机关事务、应急管理、质监、统计、气象、地震、接待、档案、地方志等工作取得新进步，妇女儿童、残联、红十字等工作扎实推进，中省直部门为地方发展做出突出贡献。

一年来，我们切实加强政府自身建设，全面贯彻落实党的十九大和习近平总书记对我省重要讲话精神，始终同以习近平同志为核心的党中央保持高度一致。认真执行市人大及其常委会的决议、决定，坚持重大事项向人大报告、向政协通报制度，自觉接受监督，102件人大代表建议、178件政协提案全部办复。坚持依法行政，乡镇以上地方政府及其工作部门全部建立法律顾问制度。抓好作风整顿活动，促进了发展环境优化。加强政府系统党风廉政建设，强化行政监察和审计监督，持之以恒正风肃纪，进一步树立清正廉洁的政府形象。

各位代表，成绩来之不易。这是市委统揽全局、正确领导的结果，是市人大、市政协有力监督、大力支持的结果，是各级各部门和全市人民团结拼搏、共同奋斗的结果。在此，我代表市人民政府，向全市人民，向各位代表、各位委员，向离退休老干部、民主党派、工商联、人民团体，向中省直单位、驻佳部队和武警官兵以及所有关心支持佳木斯发展的各界人士，致以崇高的敬意和衷心的感谢！

二、2018年政府工作面临的形势和任务

2018年，是贯彻党的十九大精神的开局之年，是改革开放40周年，是决胜全面建成小康社会、实施“十三五”规划承上启下的关键一年。深入贯彻落实党的十九大精神，切实把省委、省政府和市委的决策部署转化为生动实践，对政府工作提出了新的更高要求。更好更快推动新时代佳木斯振兴发展，需要我们进一步认清所面临的复杂形势和艰巨任务，坚定必胜信心，完成历史使命。

（一）推动新时代佳木斯振兴发展，必须对存在的困难和问题有清醒的认识。从国际看，世界经济仍处在缓慢复苏的过程中，外部环境的不稳定不确定因素增加。从国内看，经济结构性矛盾突出，实体经济困难较多，经济下行压力依然较大。从我市看，在我国社会主要矛盾发展转化的大背景下，不平衡不充分的问题更加凸显，全面建成小康社会还存在一些短板和弱项。经济总量小，经济基础薄弱是最基本的市情，2016年，全市地区生产总值仅占全省5.49%。产业结构不合理，2017年，三次产业比重28:22:50，工业短板问题突出，一季度工业经济才结束长达26个月的低迷走势，企稳回升的基础还不牢固；农业大而不强，农产品加工率仅为26.4%，农产品加工业产值与农业总产值之比远远低于全国平均值；现代服务业发展规模不大、水平和档次不高，经济开放度不够、对外贸易增长乏力。创新能力不强，创新人才偏少，经济内生动力不足。财力弱、债务重仍是制约发展的突出矛盾，增值税、所得税等反映经济活动和运行质量的收入，比全省平均水平低12.8%。民生欠账较多，城乡基本公共服务供给总量不足、水平不高、配置不均衡，公共基础设施不够完善，一些群众关心的热点难点问题没有完全得到解决，保障和改善民生压力较大。剖析这些问题的根源，虽有退出计划经济较晚、体制机制不活等历史因素，但也有思想不解放、观念不开放、工作力度不够、动力不足等主观原因。

（二）推动新时代佳木斯振兴发展，必须对把握机遇和优势有科学的判断。党的十九大报告作出了“我国经济已由高速增长阶段转向高质量发展阶段”的重大判断，中央经济工作会议把这个判断明确为“新时代我国经济发展的基本特征”。国家必将围绕高质量发展，在供给侧结构性改革、区域协调发展、乡村振兴、保障和改善民生等方面密集出台系列政策措施，既为我们振兴发展创造良好的外部环境，也为争取政策、资金、项目带来诸多机遇。特别是“中蒙俄经济走廊”建设、新一轮东北老工业基地振兴、黑瞎子岛保护与开放开发、中俄同江跨江铁路大桥即将通车、具有农业特色的国家高新区创建、抚远市重新划归我市管辖等战略机遇在佳木斯聚集叠加，丰富的资源禀赋、良好的生态环境、优越的区位条件、充足的要素保障等优势正在日益凸显、加速释放。

（三）推动新时代佳木斯振兴发展，必须对实现目标和任务有坚定的信心。佳木斯发展正处在爬坡过坎的攻坚期、有利发展的机遇期、大有可为的窗口期，形势逼人奋进，干事创业时不我待。我们要紧紧围绕市委十三届三次全会确定的“把佳木斯建成活力迸发、魅力独特、具有重要影响力的现代化区域中心城市”宏伟目标，以前所未有的危机感、紧迫感和责任感，以功成不必在我的境界和功成必须有我的担当，抢抓新机遇，应对新挑战，撸起袖子、甩开膀子、俯下身子，苦干、实干、加油干，真正让改革发展稳定的各项任务落下去，惠及百姓的各项工作实起来，加快走出质量更高、效益更好、结构更优、优势充分释放的全面振兴发展新路子。

三、2018年工作安排

今年政府工作总的要求是：全面贯彻落实党的十九大精神，以习近平新时代中国特色社会主义思想为指导，全面深入持续贯彻落实习近平总书记对我省重要讲话精神，按照中央经济工作会议、省委十二届二次全会和市委十三届三次全会要求部署，坚持新发展理念，坚持稳中求进工作总基调，坚持高质量发展，紧扣佳木斯发展实际，加快推动农业富民、工业强市、城市提档、开放兴市，努力在项目建设、深化改革、环境优化、生态建设、民生改善等方面取得新成效，奋力谱写新时代佳木斯振兴发展新篇章。

主要预期目标是：全年地区生产总值增长6.5%左右，固定资产投资增长8%左右，社会消费品零售总额增长10%左右，外贸进出口总额增长10%左右，单位GDP能耗下降3.5%左右，城乡居民人均可支配收入增长高于经济增速。

为实现上述目标，我们将重点做好以下工作:

（一）推进农业农村现代化，加快乡村振兴。把实施乡村振兴战略作为新时代“三农”工作的总抓手，坚持农业农村优先发展，推进农业大市向农业强市转变，争做新时代全国农业现代化建设排头兵，加快推动农业富民。

抓调整增效益。以推进农业供给侧结构性改革为主线，围绕市场需求，加快农业提质增效。优化种植业结构，坚持稳粮、优经、扩饲，以普及应用黄腐酸、碳基复合肥、高光效种植模式、免耕播种技术为重点，加快推广现代新型农业生产技术，全面提升科技化、机械化、标准化水平，增强绿色有机农产品原料供给能力。发展壮大现代畜牧业，引进一批大型养殖、饲料加工、畜牧产品深加工龙头企业，提升畜牧业产业化、规模化和标准化水平，新增生猪出栏能力30万头，优质高产奶牛存栏达到2万头。加大农田基础设施投入，加强生态高产标准农田建设，推进10个类别32个水利设施建设项目，增强农业综合生产能力。实施农业品牌营销战略，借助域内外电商平台，壮大农村电商群体，创新品牌的培育方式、营销技巧和推广策略，全力推进中国大米产业展览交易基地项目开工建设、完善功能、发挥作用，提升“佳木斯大米”等优质农产品知名度，让好产品真正卖出好价钱。

抓融合壮产业。以产业发展为支撑，推进农村一二三产融合，加快把食品和农副产品精深加工业打造成第一支柱产业。抓好“粮头食尾”“农头工尾”，支持益海粮油、金玛集团等产业化扩张，积极引进一批农副产品精深加工龙头企业，推动绿色食品产业规模扩张、集群发展，加快打造全国绿色有机食品中心。鼓励发展田园体验小镇、农林体验小镇等新型融合发展模式，加快现代农业产业园、科技园、创业园建设，促进农业生产、加工、物流、研发、服务相互融合，引导带动农民发展休闲农业、乡村旅游、农村电商等新产业新业态新模式。

抓改革促增收。全面启动农村集体产权制度改革，加快组建股份经济合作社，实行成员股份分红，对清理出的林地等资源性资产，加快开发利用形成新产业，让农民积极参与生产经营。完善农民和龙头企业、合作社、电商平台等利益联结机制，使农民更多分享产业增值的收益，新发展各类新型经营主体200个以上。积极探索农业社会化服务新路子，提高种业研发、农资供应、金融保险服务水平。

抓治理强基础。借助美丽乡村建设、“四好农村路”、 “厕所革命”等载体，引导城市资金、技术、人才等现代要素向农业农村流动，促进城乡融合发展。开展农村人居环境整治三年行动，加强农村垃圾、污水集中收集、无害化处理，提升村屯绿化美化净化水平。全面塑造淳朴文明的良好乡风，健全自治、法治、德治相结合的乡村治理体系，使农民安居乐业、农村安定有序。

（二）加快转型升级，提升工业经济质量效益。坚持向存量要效益，向增量要发展，向创新要动力，向园区要集聚，全力做好“三篇大文章”，打好转方式调结构攻坚战，加快推动工业强市。

双轮联动。把发挥现有企业作用和推进产业项目建设有机统一起来，推动存量和增量“两个轮子”一起转，发挥好对工业经济发展的支撑作用。做优存量。加强工业运行分析调控，抓好分类施策、定向服务和要素保障，加大对佳电股份、约翰迪尔、佳星玻璃等“老字号”企业扶持力度，不断壮大优势骨干企业。支持企业加快技术改造、新产品开发和管理创新，积极推进电机屏蔽电泵、骥驰拖拉机改造、润特超高压电缆、万龙高档再生包装纸等项目建设，加快推动装备制造、食品、造纸等产业向中高端迈进。实施中小企业成长工程，梯度培育，精准扶持，促进中小微企业向“专精特新”发展，推动更多企业向规上转变。做大增量。坚持引资引技引智相结合，大力引进一批知识技术密集、物质资源消耗少、成长潜力大、综合效益好的大项目好项目，加快发展新材料、生物技术、新型建材、电子信息等战略性新兴产业，重点推进高性能聚芳酯特种树脂材料、生物科技新型除草剂、山梨醇、燃料乙醇、聚兴碳基复合肥、汽车用铝型材等项目建设，积极争取与央企合作的电子信息产业园、新型建材产业基地、光伏发电等项目尽早落地，形成多领域、多产业、多增长点的发展格局。

创新驱动。把创新作为引领工业发展的第一动力，加快培育高新技术企业，提升工业核心竞争力。深入实施国家高新技术企业双倍增三年培育计划，扩大“院士专家佳木斯行”活动成果，深化与中国科学院长春分院合作，充分发挥域内高等院校、科研院所等平台作用，加速推进科技成果转移转化，产学研合作嫁接生成一批；加快冬梅豆粉、葵花药业等企业技术中心建设，增强企业自主创新能力，传统企业升级提升一批；鼓励和推广创新工场、虚拟孵化器等新型孵化模式，推动“孵化+创投”等模式创新，加快各类科技孵化器和众创空间的建设，创新创业培养成长一批。

园区带动。举全市之力创建具有农业特色的国家高新区，带动工业总量扩张。增强高新区承载功能，完善路网、污水处理、电力、铁路专用线等基础设施，推进综合服务大楼等配套设施建设，加快向园区引进金融服务、科技服务、电子信息、产品检测、云计算、孵化器等项目和机构。坚持以园招商、以商招商、精准招商，加快引进落地一批与高新区产业定位契合、辐射带动力强的大项目，积极推进七彩毯业、天和纺织等老企业退城进园，推动项目向园区集中、产业向园区集聚。完善项目包保机制，加大对在建项目和已落户企业服务保障，帮助企业解决融资等难题，全力支持泉林一期达产达效，加快二期工程建设，确保年内部分投产。各县（市）区要完善园区产业功能定位，加强分工合作，形成错位、协同、融合发展格局。

（三）发展现代服务业，优化城市综合功能。围绕推进区域中心建设，聚焦重点，精准发力，提升现代服务业在城市经济中的主体地位，加快推动城市提档。

突出便捷畅通，加快建设区域交通中心。强化枢纽地位，完善内联外通的现代综合交通网络，开工建设公铁联运客运枢纽站，加快佳木斯站综合改造和高铁西站市政配套设施建设，确保哈佳快速铁路按时开通，牡佳客运专线加快建设；实施路桥畅通工程，加快城市重点路桥建设，改造升级佳木斯至汤原、桦川、横头和胜利路西段等城市周边路网，启动建设松花江二桥；推进佳木斯机场迁建前期工作。启动新一轮城市总体规划修编和空间规划编制，抓好生态修复、城市修补工作，完善城市园林、水系等景观体系，加快海绵城市、智慧城市建设，不断增强城市承载能力。

突出辐射带动，加快建设区域商贸物流中心。加快商贸龙头企业高端化、品质化、特色化转型，持续提升骨干商圈的影响力。启动中央公园项目立项，推进佳木斯铁路物流基地、广源物流迁建重组等项目建设，推广电商营销模式，加快形成与电子商务相配套的互联网服务和快递网络，增强区域物流集散能力。

突出服务优化，加快建设区域健康医养中心。依托佳大一附院、市中心医院等医疗品牌的影响力，加强重点专科建设，强化疾病预防控制和紧急救治能力，构建覆盖城乡、辐射周边的优质医疗服务体系。支持有条件的二级及以上医疗机构开设老年病科，鼓励社会资本参与建设医养结合机构，全面提升医养结合服务水平。

突出平台构建，加快建设区域科技创新中心。以市校共建“环佳木斯大学知识经济圈”为载体，加大政策、资金、人才等要素资源倾斜力度，支持佳木斯大学科技企业孵化器升级为国家级孵化器，加快建立技术创新市场导向机制、科技成果转化机制、市校协同创新机制，努力构建有效连接企业、科研院所、科技服务机构、金融机构的创新创业平台。启动佳木斯工业技术研究院建设，加快形成具有共性技术及关键技术研发、成果转化、企业孵化、技术服务和人才培养“五位一体”的协同创新平台。

突出产品创新，建设区域文化旅游中心。借助夏季整体生态优势和冬季冰雪资源，充分融入抗联、东北小延安、知青、赫哲等特色文化，积极引进战略投资者，全面加强景区景点等旅游基础设施建设，深度开发华夏东极、界江界岛、赫哲民俗、红色文化、生态农业、森林湿地、赏冰乐雪等区域文化旅游产品，打造自然风光游、历史文化游、异国风情游等精品旅游线路，加快推进域内旅游产品集成营销、规模经营，景点景区连点成线、扩线成面，形成全域旅游发展新格局。

突出质量提升，加快建设区域教育中心。科学配置和扩展优质教育资源，全面提升高等教育、职业教育、基础教育、特殊教育、民族教育的办学质量，鼓励引导民办教育健康发展，切实加强教育国际交流合作，推进各类教育质量全面提高，切实满足城市及周边对教育的多样化需求。

（四）扩大全方位对外开放，提高开放型经济水平。积极融入国家“一带一路”“中蒙俄经济走廊”建设，在我省“打造一个窗口、建设四个区”中找准定位，牢牢把握中俄同江跨江铁路大桥建设和黑瞎子岛保护与开放开发机遇，加快形成特色鲜明的开放新格局。

加强口岸通道建设，使功能强起来。全面提升跨境基础设施建设水平，打造“中蒙俄经济走廊”重要枢纽。加快黑瞎子岛保护与开放开发，启动编制国际旅游岛建设规划，争取开通乌苏镇至哈巴扎伊姆卡旅游口岸，力争中俄同江跨江铁路大桥年内竣工通车，推动黑瞎子岛公路口岸和同江铁路口岸申报，加快形成以佳木斯城区为中心，同江和抚远为两翼，三点联动、互补发展的格局。积极推进松花江、黑龙江下游航道疏浚和口岸改造等项目建设，提高航道通航等级和水运能力。推进佳木斯至哈巴罗夫斯克航线向俄腹地延伸、佳木斯至比罗比詹国际汽车直达运输线路向哈巴罗夫斯克延伸。

打造对外开放平台，使外向型产业活起来。以对俄开放型经济区和产业园区建设为支撑，促进境内外产业联动、上下游产业衔接，提升经济发展的外向度。积极申报和创建佳木斯综合保税区、黑瞎子岛中俄跨境经济合作区、同江国家级边境经济合作区，大力推进保税仓库、专业交易市场、跨境电商等项目建设。科学规划中俄同江跨江铁路大桥桥头经济区功能和产业布局，加快国储能源储运园区、中林同江木材产业园等境内外园区建设，谋划辟建佳木斯空港经济区。依托“哈佳双同”产业集聚带建设，加强引俄木材、石油、天然气、金属、矿产等资源能源合作开发，大力培育外贸主体，支持有实力的企业、有闯劲的企业家“走出去”，建设农机、机电、建材、轻纺、食品、果蔬等外向型出口加工基地，做好旅游、商贸、科技、文化等相关产业对接，全力培育跨境产业链。创新对外经济合作方式，加强对俄文化、教育、科技等人文交流，推动沿边开放向欧洲腹地延伸，深化与农垦、森工等驻佳单位合作共建，全面推进与广东省中山市对口合作。

优化通关环境，使效能提起来。加强与俄方口岸的通关合作，密切口岸、内地协作机制，全面实施“信息互换、监管互认、执法互助”和“一次申报、一次查验、一次放行”的关检合作模式，加快构建便捷快速的进出口、过境、转运和通关货物大平台，推进口岸查验工作前推后移，加大直通放行力度，实行报关、报检预约通关制度和上门查验服务措施，提高通关效率，降低通关成本，优化通关环境。

（五）深化基础性关键领域改革，激发内生发展动力。突出问题导向，坚持靶向原则，打好关键领域改革攻坚战，坚定不移推进改革向纵深发展。深化经济体制改革。以经济体制改革为主轴，牵引和带动其他领域改革，使各方面改革协同推进、形成合力。推进财政体制改革，加快市、区事权与支出责任划分改革，深化政府采购、财政投资评审管理改革，推动绩效预算改革全覆盖。推进税收体制改革，巩固和扩大营改增成果，进一步减轻企业税收负担，营造简洁透明、更加公平的税收环境。推进金融体制改革，促进金融机构突出主业、下沉重心，大力发展普惠金融，健全地方金融监管体制，未完成改制的富锦、桦南、桦川、郊区等4个农村信用社，力争年底前达到农村商业银行组建条件。推进投融资体制改革，推行企业投资项目承诺制，支持社会资本参与基础设施建设和公共服务，推动企业扩大直接融资比重。深化供给侧结构性改革。按照高质量发展要求，深入推进“三去一降一补”，全面完成省下达的去产能任务，稳妥做好职工安置和债务处置工作。加强房地产市场调控，加大消化商品房库存力度。强化政府债务和企业资金链管理，盘活政府非办公类资产，将更多的公共资源配置到生产领域。千方百计降低企业制度性交易成本、税费成本、融资成本。深化“放管服”改革。坚定不移推进这一“牵一发动全身”的改革，加快政府职能转变。全面实施市场准入负面清单制度，抓好行政审批流程再造，完善并联审批工作机制，大力推行“只见一次面审批、网上不见面审批”，让信息多跑路、群众少跑腿。持续开展“减证便民”行动，推动商事制度改革扩容升级。创新监管方式，加强事中事后监管，提高“双随机一公开”精细化水平。完善公共交易平台建设，规范公共资源交易活动，提高市场化招标质量效率。深化民生领域改革。牢牢把握改革的根本出发点，突出民生导向，切实抓好教育体制、医药卫生体制、社会保障体制、收入分配制度、住房制度等改革，着力织牢“民生网”，使各项民生改革在实施过程中相互促进、在改革成效上相得益彰，进一步增加公共产品有效供给，推进社会公平正义。

（六）保护生态环境，依法治理环境污染。打好污染防治攻坚战，使佳木斯天更蓝、水更清、山更绿、生态环境更优美。

解决环境突出问题。开展减霾卫蓝行动，严格落实“大气十条”，打好“减煤、控车、抑尘、治源、禁燃、增绿”组合拳，妥善应对重污染天气，确保城市空气质量优良天数稳中有升。开展治污净水行动，严格落实“水十条”，深入落实河长制，建立湖长制，加大流域环境监管、饮用水源保护和生活污水治理力度，加强水源地保护和规范化建设，加快黑臭水体整治工程建设，推动水环境质量持续好转。开展保土护田行动，严格落实“土十条”，编制我市《土壤污染治理与修复规划》，强化土壤污染管控和修复，加强农业面源污染防治，开展农用地污染详查点位监测、尾矿库企业环境风险评估等工作，采取工程、农艺、生物等综合措施提升黑土耕地质量，让黑土地得到永续利用。

加强资源节约循环利用。严格落实能源和水资源消耗、建设用地等总量和强度“双控”，推行企业循环式生产、产业循环式组合、园区循环式改造，加快形成有利于资源节约和高效利用的空间格局、产业结构、生产方式、消费模式。加大秸秆综合利用力度，树立秸秆是资源的新理念，积极引进社会资本，提高秸秆综合利用率。加快建设北京三聚环保公司利用秸秆生产碳基复合肥项目，多渠道增强秸秆消化能力，提高秸秆综合利用规模化、产业化水平。

强化生态保护制度落实。完善体现生态文明要求的目标体系、考核办法、责任追究机制，健全环保信用评价、环境损害赔偿等制度，加快划定生态保护红线。加大中央环保督察反馈意见整改力度，全面开展领导干部自然资源离任审计，严肃问责在环境问题上履职不力的干部。对大气、水、土壤、自然保护区开展常态化执法检查，对恶意违法排污零容忍、顶格处理，形成有力震慑，用最严格的制度守护好绿水青山。

（七）坚持标本兼治，持续优化发展环境。持之以恒、久久为功，打好整顿作风优化发展环境攻坚战，为经济发展提供优质的环境保障。

优化法治环境。严格执法资质，完善执法程序，建立健全执法公示、执法全过程记录、重大执法决定法制性审核等制度，着力解决职责交叉、多头执法、重复处罚等问题。深入推进“七五”普法，引导广大干部群众尊法学法守法用法。突出惩治破坏市场经济秩序犯罪，依法严厉打击和查处各类扰乱市场秩序、侵害消费者权益、影响城市形象的违法违规行为。

加强诚信建设。健全政务诚信约束和问责机制，加大对危害群众利益、损害市场公平交易等政务失信行为的整治力度，进一步提升政府公信力，引领商务、社会、司法等重点领域诚信建设。加强诚信教育，开展诚信公益活动，推广普及信用知识，倡导良好诚信风尚。建立健全守信激励、失信惩戒机制，开展诚信专项治理，依法曝光社会影响恶劣、情节严重的失信案件，坚决打击各种不良信用行为，使守信者处处受益、失信者寸步难行，营造良好社会信用环境。

深化作风整顿。坚决摒弃 “三个坏把式”，持续整顿“五个坏作风”，巩固扩大作风整顿成果，推动作风整顿向基层延伸。聚焦不作为、慢作为、乱作为等突出问题，紧盯关键岗位、重点部门、服务窗口和关键人，集中开展专项整治行动，坚决查处败坏作风、破坏发展环境的人和事。全面推行政务公开，强化内部流程控制，健全首问负责、岗位责任、一次性告知、限时办结、服务承诺、投诉举报等工作制度，深入推进“四零”承诺创建，切实解决“门好进、脸好看、事难办”问题。

（八）全力保障和改善民生，不断增强群众获得感。坚持以人民为中心的发展思想，多办实事好事，多解难事忧事，使振兴发展成果更多更公平惠及于民。

坚决打好精准脱贫攻坚战。聚焦坚中之坚，压实落靠各级责任，加大扶贫领域作风整治和督查整改力度，把精准要求贯穿全过程，从严从实把好精准识别关、精准施策关、精准退出关，保证现行标准下的脱贫质量，确保国贫县总体退出工作经得起国省评估检查，真正让群众满意。主攻产业扶贫软肋，精准确定主打产业，发挥新型主体带动作用，用好电子商务渠道，积极发展光伏产业，带动贫困人口长期稳定脱贫致富。补齐“三保障”和饮水安全短板，补足基础设施建设和公共服务弱项，抓好金融扶贫和驻村帮扶，注重扶贫、扶志、扶智相结合，进一步提高贫困群众自我发展能力和脱贫致富内生动力，切实做到脱真贫、真脱贫。

持续提升社会保障水平。全力抓好城镇转移就业职工、农村剩余劳动力、高校毕业生、复员退伍军人等重点群体就业，城镇新增就业4.67万人，零就业家庭动态为零。推进全民参保计划，实施统一的城乡居民医疗保险制度和大病保险制度，加快社保卡采集发放应用，努力实现社会保险法定人群全覆盖。统筹推进扶老、助残、救孤、济困、留守妇女儿童关爱等福利和慈善事业发展。

优化城乡公共服务。坚持优先发展教育事业，优化教育布局，推进县域义务教育发展基本均衡，实施高中阶段教育攻坚计划，稳妥推进高考改革，创建国家级示范性骨干职教集团。推进健康佳木斯建设，加强医联体建设和家庭医生签约服务，加快构建全人群的健康管理体系、全生命周期的服务体系和全方位城乡医疗保障体系。推动文化体育事业繁荣发展，加大公共文化设施和体育基础设施建设力度，广泛开展群众性文体活动，推动文化精品创作和精品演出，加强非物质文化遗产保护工作。

加强和创新社会治理。完善安全生产责任体系、预防体系和监管体系，确保安全生产形势持续稳定。创新群众来访接待和矛盾调处工作机制，集中力量化解信访积案。保障军人合法权益。创新城市管理方式，推动城市管理向城市治理转变。积极创建国家食品安全示范城市，持续提升食品药品安全治理能力和保障水平。加强立体化社会治安防控体系建设，严厉打击危害公共安全等各类违法犯罪，不断提升群众安全感和满意度。

办好办实民生实事。想群众之所想，急群众之所急，既尽力而为，又量力而行，重点办好10件民生实事。①解决群众反映强烈的供热问题，加大热源建设和老旧管网改造力度，强化对供热企业的管理与监督，切实为群众提供温暖的生活居住环境。②继续加大“回迁办证”工作力度，确保年底前回迁安置动迁居民3000户，进一步解决已入住居民不能办理不动产权证问题。③大力改善群众居住条件，让棚户区居民出棚上楼，新开工改造棚户区7898套，新增廉租房租金补贴1000户，改造农村危（草）房5000户。④强化物业管理，提高物业服务水平，建立健全物业企业准入退出机制，加大解决弃管楼问题力度，维修改造老旧小区楼房100栋。⑤为群众提供优质公共服务，新建4个1000平方米集养老服务、社区矫正、康复理疗、文体娱乐等功能于一体的省级示范社区办公用房，年底前，建成使用光荣院、东兴养老养护中心和荣军养老中心，完成敖其镇殡仪馆改扩建工程。⑥办好人民满意教育，增加普惠性幼儿园比例，解决好中心城区热点学校“大班额”问题。⑦完成市中心医院改扩建以及市中心血站实验室、市儿童医院和妇幼保健院续建项目，确保年底前投入使用。⑧实施“厕所革命”，改造农村居民室内厕所8000个，新建改建城镇公厕100座，旅游景区厕所全部达到国家规范标准。⑨推进城市交通畅通，打通断头路，完成和平街、建圃东街南段、通江街立交桥、通园巷立交桥等工程，开工建设兴国路、科技大道立交桥等重点工程，加大主次街道维修改造力度，开展常态化道路交通秩序整顿，重点治理校园周边交通拥堵，新增停车泊位1000个、电动公交车65台，让百姓出行更顺畅更安全。⑩实施佳木斯机场保障性功能扩建，增加2个停机位、1个联络道，提高航空运输保障能力。

四、切实加强政府自身建设

以更加坚定的政治立场、更加进取的精神状态、更加务实的责任担当、更加执着的为民情怀，全面提升政府执行力、凝聚力和战斗力。

（一）强化“四个意识”，忠诚履职。深入学习贯彻落实党的十九大精神，坚决维护以习近平同志为核心的党中央权威和集中统一领导，始终在思想上政治上行动上同以习近平同志为核心的党中央保持高度一致。不折不扣贯彻落实省委、省政府和市委各项决策部署，确保政令畅通、令行禁止。

（二）强化法治思维，依法行政。严格依照法定权限和程序履行职责，完善权责一致、执行顺畅、监督有力的行政管理体制。履行重大决策法定程序，严格执行“三重一大”制度，促进科学决策、民主决策。自觉接受市人大及其常委会法律监督、工作监督和市政协民主监督，广泛听取各民主党派、工商联、无党派人士和人民团体的意见，主动接受社会和舆论监督，让权力在阳光下运行。

（三）强化工作落实，务实高效。围绕适应高质量发展要求，加快提升能力素质，建设学习型、创新型、实干型的政府干部队伍。完善抓落实的制度链条，健全执行、监督、考评、奖惩等工作机制，铆紧各方责任，层层传导压力。任何工作都要抓早抓细抓实，抓责任人抓节点抓效果，一条心、一股绳、一个劲，推动各项工作干在实处、走在前列、争创一流。

（四）强化廉洁从政，风清气正。严格落实全面从严治党各项要求，以永远在路上的韧劲反“四风”、转作风，严厉整肃庸政懒政怠政行为。巩固“两学一做”学习教育常态化制度化成果，开展好“不忘初心、牢记使命”主题教育。健全廉政风险防控机制，加大重大政策措施落实情况跟踪审计，突出公共财政审计监督，严控“三公”经费支出，严防权力失控、决策失误、行为失范。

各位代表，新时代要有新气象，更要有新作为。让我们更加紧密团结在以习近平同志为核心的党中央周围，以习近平新时代中国特色社会主义思想为指导，在市委的坚强领导下，凝心聚力，真抓实干，奋力谱写新时代佳木斯振兴发展新篇章!
